# Geopolymer Foams—Will They Ever Become a Viable Alternative to Popular Insulation Materials?—A Critical Opinion

**DOI:** 10.3390/ma14133568

**Published:** 2021-06-25

**Authors:** Michał Łach

**Affiliations:** Institute of Materials Engineering, Faculty of Materials Engineering and Physics, Cracow University of Technology, Jana Pawła II 37, 31-864 Cracow, Poland; michal.lach@pk.edu.pl; Tel.: +48-12-6283-454

**Keywords:** geopolymers, insulating materials, alkali-activated materials

## Abstract

Over the last several years, there has been a large increase in interest in geopolymer materials, which are usually produced from waste materials, and their applications. The possibilities of application of geopolymers seem to be unlimited, and they are used in almost all fields of technology. Their use as insulation materials appears promising due to their complete nonflammability and excellent strength. However, one limitation is their complex manufacturing process and lack of stability of the obtained geopolymer foams as well as difficulties in achieving such good insulation properties possessed by polyurethane foams, polystyrene, and wool. Hundreds of studies have already been performed on insulating geopolymer foams and various types of foaming agents, and their authors reported that foamed insulating geopolymers had a density starting from 200 kg/m^3^ and thermal conductivity from 0.04 W/mK. However, the repeatability of the obtained results on an industrial scale is questionable. It is still a challenge to obtain a geopolymer material with comparable properties as conventional insulation materials and to overcome the barriers associated with the successful implementation of geopolymer material as insulation in buildings and other applications on a mass scale. This paper provides a comprehensive review of the methods used for the production of foamed geopolymers and the best parameters obtained, as well as a summary of the most important information reported in the scientific literature. It also presents the results of a critical analysis of the feasibility of implementing this technology for mass deployment. In addition, the problems and limitations that are most often encountered with the implementation of geopolymer technology are discussed.

## 1. Introduction

**Figure 1:**
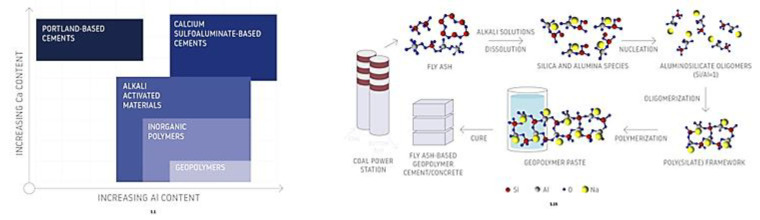
Process used for making geopolymers (adapted from [6]) and a schematic summary of the chemical relationships between geopolymers and other binders (adapted from [9]).

Geopolymers are materials defined as inorganic polymers [1,2,3]. They are usually produced from waste aluminosilicates (various types of fly ash, slag, or waste resulting from ceramic, metallurgical, and power industries) [4,5] or natural materials such as volcanic tuffs and metakaolin [6], clays [7], and other sources of aluminosilicates [8]. Resin-based materials that are used for injection to achieve soil stabilization, among others, are often mistakenly referred to as geopolymers. Figure 1 shows the geopolymers that can be compared to other binders, by illustrating the process used for making geopolymers as well as providing a schematic summary of the chemical relationships between geopolymers and other binders.

**Figure 2:**
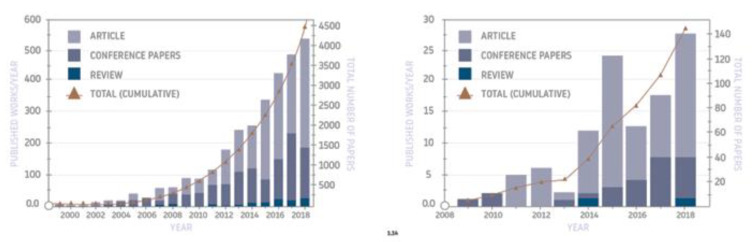
Summary showing the number of publications in recent years on geopolymers (**left**) and foamed geopolymers (**right**) (based on a recent review [10]).

Geopolymers have been known for several decades (although under the name “geopolymers”—since the time of Prof. Davidovits—the 1970s), but in recent years there has been a significant increase in interest in these materials for specific applications, which has also been manifested by an increase in the research related to them. As shown by a recent review of publications [10] (Figure 2), the last few years have seen a remarkable increase in scientific papers in the area of both geopolymers in general and foamed geopolymers in particular. This demonstrates the importance of these materials and the prospects of the scientific community on their application as an alternative to other conventional materials.

**Figure 3:**
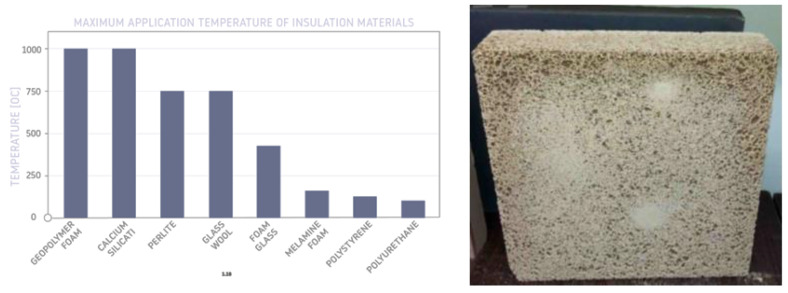
Maximum application temperature of some insulation materials, adapted from [12] and an example of foamed geopolymer panel (50 cm × 50 cm) (own research).

For many years now, it has been well established that the potential uses of geopolymers are extensive. One area of the application of alkali-activated mineral materials and geopolymers can be the production of foamed materials with low thermal conductivity and high fire resistance properties. Foamed inorganic polymers (geopolymers) have excellent mechanical strength and fire safety [11]. They exhibit the highest fire resistance, compared to other common insulation materials. A comparison of the maximum working temperatures of insulation materials is presented in Figure 3.

The problem that engineers have been struggling with for several decades is how to effectively insulate buildings to minimize energy losses. When designing insulation materials, apart from their effectiveness, one should also take into account: safety of use, non-flammability (or its limitation), easy application method, no negative impact on the environment. Experience shows that this is not always an easy task to achieve and there are often very negative effects when using unsuitable materials. Years ago, asbestos was one of the best temperature-resistant materials and often used for high-temperature sealing, insulation and construction in general. Unfortunately, its negative impact on human health is now known. At present, insulating materials, such as polystyrene, mineral wool, glass wool, etc., are very popular. Recently, PUR and PIR foams applied by spraying on various surfaces have also become popular. Unfortunately, each of these materials has some drawbacks and they are used where there is a certain compromise between their advantages and disadvantages. Foamed geopolymers seem to be the best solution to eliminate the disadvantages of other materials.

After application, commonly used polyurethane foams are harmless to health and do not emit any harmful substances. However, be careful when working with them. Isocyanates are very toxic and bind to compounds that are part of living organisms: water, proteins, acids, etc. Low molecular weight polyisocyanates are the most harmful. The main effects of their action on the human body are irritation of the mucous membranes of the respiratory tract, eyes and skin. The most common symptoms of poisoning are: lacrimation, redness and burning of the conjunctiva and nasal mucosa, scratching the throat, coughing, shortness of breath, pressure behind the breastbone and chest pain.

Problems related to the use of polystyrene are mainly its problems with subsequent disposal and flammability. It is accused of this technology that polystyrene boards glued to the facade can transfer fire to higher floors. At present, the use of polystyrene for thermal insulation of buildings is very much limited (attempts are being made to limit). The geopolymer technology is completely resistant to fire, even at temperatures above 1000 °C. It is also completely harmless to the environment and to the people involved in the application of these materials. No harmful substances are emitted during the production and application of geopolymers.

Due to low weight of geopolymer foams, these materials have found many applications in industries as well as in construction [12]. One of the first described materials of this type had the trade name TROLIT [12] (in addition to the geopolymer–wood fireproof panels first implemented by Prof. Davidovits). TROLIT was a type of inorganic geopolymer foam made using three main components: (1) solid components that are a source of silicon and aluminum, usually obtained as by-products from industrial processes involving high temperatures; (2) a liquid activator consisting of aqueous solutions of alkali metals; and (3) expanding agents, such as peroxides and perborates. This material was characterized by unique properties that few scientists had been able to achieve before, but only on a laboratory scale. The material had a thermal conductivity of 0.037 W/mK, which is close to that of polystyrene.

Similar parameters have been reported by other authors. For instance, Davidovits reported a material obtained by the fabrication of a porous material with a geopolymer framework using blowing agents such as hydrogen peroxide (H_2_O_2_) or sodium perborate (NaBO_3_) [1]. This work confirmed the possibility of producing geopolymer foams with a density between 0.2 and 0.8 g/cm^3^, an apparent heat resistance up to 1200 °C, and a minimum thermal conductivity of 0.037 W/mK. Using the same principle, Bell and Kriven [13] developed various foams by the addition of H_2_O_2_ and aluminum powder and investigated their properties. Vaou and Panias [14] succeeded in reducing the thermal conductivity coefficient of foams to 0.030 W/mK using perlite. However, these parameters are not universally reproducible by most researchers, and several recent scientific papers and industrial experiments showed that these parameters are very difficult to achieve—although not impossible. This lack of reproducibility and difficulty in obtaining such high insulation parameters is an important barrier to mass production.

It should also be noted that the parameters of foamed geopolymers are often compared to that of polystyrene but not expanded polystyrene, which is the most popular insulation material [15,16]. Compared to expanded polystyrene, foamed geopolymers with a density of, for example, 0.07 W/mK are not considered attractive anymore.

**Figure 4:**
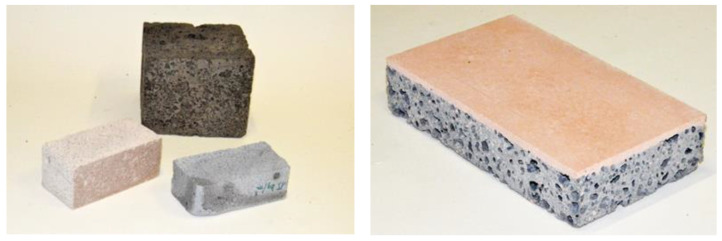
Examples of foamed geopolymer materials based on various waste materials (source—own research).

Currently, various laboratories around the world are working to develop foamed geopolymers with the best possible insulation performance. However, there are no standard procedures and process parameters for obtaining geopolymer foams. For developing these foams, the parameters must be selected individually depending on the available local raw materials. It has been found that a good relationship exists between the heat conductivity coefficient and the density of the produced materials. To improve (reduce) the thermal conductivity coefficient, it would be necessary to decrease the density of the material (increase the degree of foaming) by increasing the amount of foaming agent [17,18]. However, reduction of density is not always possible as it will cause a reduction in strength parameters. In many applications, mechanical strength plays an important role. Two- or three-layer partitions are increasingly considered, in which foamed geopolymers can be used for insulation and construction (e.g., for masonry elements designed for buildings up to two stories). Examples of layer brick and geopolymer foam elements produced from metakaolin, fly ash, and slag are shown in Figure 4.

Geopolymer foams are characterized not only by good thermal properties but also by very good acoustic insulation [19].

Review articles on foamed geopolymers and alkali-activated materials are scarce [10,20,21,22]. In the available papers, the authors mainly focus on determining the influence of a particular parameter of geopolymerization on the properties of the obtained foams. However, the usefulness of this technology and the real possibilities of its use on a mass scale have not been analyzed. The thermal conductivity values recorded by the authors of different studies differ considerably from that of popular mass insulation materials used in construction. Moreover, a number of technological problems prevent the real-life applications of such materials.

Foam geopolymer materials based on fly ash can be an interesting alternative to currently popular materials such as polystyrene, mineral wool, and glass. They are a non-flammable material characterized by relatively good insulation parameters. Tests [23] showed that at a density of 375 kg/m^3^, the geopolymer foams have a compressive strength of about 0.68 MPa and in this case, the bending strength was 0.45 MPa. As the density decreases, the strength properties decrease. This subject requires constant research and development due to difficulties in controlling the foaming process and due to the problem and difficulties associated with the stabilization of geopolymer foams. Economic aspects should also be considered, and expensive additives will not always be cost effective. The geopolymer material, however, is particularly interesting nowadays due to the closed-loop economy as well as due to its incredible thermal resistance properties. Perhaps this material will soon be implemented on an industrial scale as an alternative to the currently used insulation materials in construction.

This paper presents the results of recent research related to achieving the best possible insulation performance in foamed geopolymers and critically addresses the prospects for the mass production of these materials. The author of this paper has over 10 years of experience related to research and implementation trials of geopolymers. He is a co-founder of a company involved in the commercialization of geopolymer materials.

## 2. Foaming Technology and Raw Materials

The applications of foamed geopolymers are very extensive. Due to their unique fire resistance and mechanical strength, these materials can be used in both common thermal insulation of buildings and specialized industrial applications (at operating temperatures above 1000 °C). A key factor here is that similar thermal conductivity properties can be achieved when manufacturing such materials. However, as can be seen from numerous scientific studies, this is a challenging task due to the fact that foamed geopolymers are highly unstable even before curing. Beyond the standard possibilities of using foamed geopolymers as thermal insulation in construction [9,23,24,25,26,27,28,29,30,31,32,33,34,35,36,37,38,39,40,41], they can be applied as hybrid organic–inorganic foams for the removal of metal ion contaminants from wastewater [24], making foam filters with the addition of lignocellulose from sawdust, and for the filtration of dyes and wastewater [42].

In addition to the issues related to stability, sagging often occurs during the production of geopolymer foams. However, this can be prevented by adding, for example, foam stabilizers. These stabilizers can be added as surfactants or in the form of Portland cement or lime [43]. Fresh geopolymer foams have been stabilized by adding nonionic surfactants such as Tween 80—a polyoxyethylene 20 sorbitan monooleate (C_64_H_124_O_26_; VWR BDH Prolabo) and Triton X-100—a polyethylene glycol tertoctylphenyl ether (C_14_H_22O_, (C_2_H_4_O)n, n = 9–10; Sigma-Aldrich, Poznań, Poland) [36]. Pork lard or butter, for example, was used as a surfactant [44]. Olive oil has also been considered, as well as agents, such as Sika Lightcrete 02 (containing, e.g., 40 wt% solution of fatty acid), amide, and sodium salt of C_14_–C_16_ sulfonic acid in water [36]. Furthermore, chemicals such as polyacrylic acid (Dolapix CE-64) are added to reduce viscosity [36]. Good results are also obtained with calcium stearate [45].

Various methods for the production of porous geopolymers have been presented in previous publications [23,43,46,47,48,49,50,51,52,53,54,55,56,57,58,59,60].

Fly ash originating from coal combustion [36,61], diatomite [62], metakaolin [36], and palm oil fuel ash [12] are usually used to produce foamed geopolymers. Calcium phosphate biomass ashes combined with metakaolin in a ratio of 1:1 have also been used for production. When such materials were foamed using H_2_O_2_ at 5% by weight, foams with a density of about 310 kg/m^3^ and thermal conductivity of 0.073 W/mK were obtained. The compressive strength of the obtained materials was estimated at 0.6 MPa [59]. To obtain optimal performance of geopolymer foams, metakaolin and fly ash are often used in combination. The authors of the cited paper proved that the most optimal composition for this kind of raw material is 62.5% of metakaolin, 12.5% of fly ash, and 25% of activator. Such a composition was foamed using H_2_O_2_ in a ratio of 1:1.5:2%, which resulted in geopolymer foams with a density of 225–506 kg/m^3^ and thermal conductivity of 0.07–0.12 W/mK. This study also showed that the porous structure of foams is very much affected by the use of surfactants [60]. Natural soil can be used as a precursor for geopolymer formation [45]. Various additives, such as coke dust waste, can be added to improve the mechanical strength of geopolymers [63], but this can affect their thermal conductivity. Fibers, especially waste natural fibers, can be used for reinforcement to strengthen foamed geopolymers. However, further efforts are needed to develop effective technologies for the use of natural fibers in geopolymer foams. Foamed geopolymers and geopolymers reinforced with natural fiber composites are often associated with many challenges related to, among others, fiber adhesion, foam stability, variable chemical compositions, and mechanical processing of fibers [22].

Aluminum powder or elemental silicon-containing materials (silica dust, FeSi, or SiC) can be used as gas-generating agents. In an alkaline environment, Al or Si reaction takes place followed by the release (generation) of hydrogen gas [43]. Sodium hypochlorite (NaOCl) can also be possibly used as a foaming agent [52], as well as the most commonly described hydrogen peroxide and sodium perborate in amounts of, for example, 1, 2, or 3% [50]. Furthermore, increased porosity can be achieved by in situ formation of surfactants by saponification of oil in an alkaline environment [46]. Rice husk can also be a potential foaming agent [55]. Metakaolin-based geopolymers foamed using H_2_O_2_ in concentrations ranging from 0.25% (*w/w*) to 1.25% (*w/w*) had compressive strengths of 6 MPa (for 0.25% H_2_O_2_) to 0.36 MPa (for 1.25% H_2_O_2_) and thermal conductivity of 0.298 W/mK (for 0.25 wt% H_2_O_2_) to 0.172 W/mK (for 1.25 wt% H_2_O_2_) [56]. The use of aluminum powder as a foaming agent in concentrations of 3.6% and 9% allowed obtaining foamed geopolymers with a density below 700 kg/m3 and thermal conductivity of <0.1 W/mK [64].

**Figure 5:**
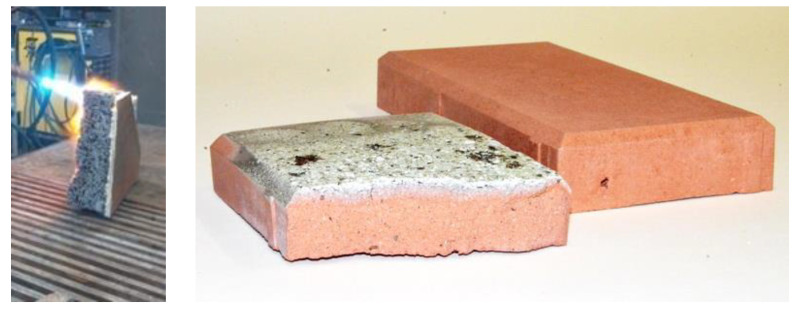
Geopolymer materials tested by oxy-acetylene torch (temperature > 3000 °C) (source—own research).

Porous geopolymers show an increase in compressive strength after exposure to temperatures of 1000 °C. This is attributed to an increase in polycondensation and sintering reactions at such temperatures. It was found that the fire resistance characteristic of geopolymers strongly depends on their chemical composition. One of the important parameters influencing the strength of porous geopolymers subjected to high temperatures is the Na_2_SiO_3_/NaOH ratio. The highest strengths of these materials are obtained at a ratio of 3.5 [8]. Fire resistance also improves with an increase in the content of, for example, K_2_O [12] (Figure 5).

## 3. Limitations Related to the Implementation of Geopolymer Technology and Other Technical Problems

An important advantage of geopolymer materials is that they can be produced from waste materials originating from various industrial processes. However, this also limits the wide application of these materials because it is difficult to regulate the production process on a mass scale due to the variability of the raw material. Research results obtained from different laboratories around the world are actually useful only for a specific type of waste and can be translated to a mass scale only if an appropriate technological regime is applied. For a long time, it has been known that geopolymer technology is very sensitive to changes in the chemical and phase composition as well as in process parameters. Therefore, controlling the process used for the formation of geopolymer foams is challenging, since maintaining the stability of foamed structures is very difficult. The repeatability of individual results on the basis of obtained samples is also poor. Geopolymerization and foaming is a complex technology to be implemented on an industrial scale, and there is no evidence of mass-scale production of this type of product. Although numerous attempts are made in laboratories worldwide, the current state of technology does not allow for effective mass-scale implementation of geopolymer production and the use of geopolymer foams interchangeably with popular insulation materials such as expanded polystyrene, polyurethane foams, and wool.

Despite the publication of thousands of scientific papers, the information about the technology of geopolymer production remains limited. Scientific publications only provide recipes or proportions of activating components for a specific raw material, which is usually fly ash obtained from a specific coal (or other materials) combustion plant. Reproducibility of the results is only possible with the use of the same ash obtained from nearly the same period of time—the composition and morphology of ash in flue gas-cleaning installations change almost every day. On the other hand, in patents, the authors claim solutions involving the use of ashes with chemical compositions within a certain range and activating additives also within certain ranges.

As described in a paper [65], the Federal Highway Administration (FHWA) prepared a technical paper on the use of geopolymer concrete as a part of its concrete pavement technology program [16]. This document outlines the following current limitations associated with the use of geopolymers:

- Risks associated with health and safety and the use of alkaline activating solutions
- Processing of high-alkalinity solution and associated energy consumption and greenhouse gas generation
- Sensitivity to temperature
- Need of strictly controlled curing at elevated temperatures

### 3.1. Changes in Raw Material Prices

A very important problem that affects the implementation of geopolymer technology is that most scientific studies concern the use of fly ash and slag, which are running out or increasing in costs, due to the environmental policy and the avoidance of coal-fired power generation. Therefore, there is a need to search for new alternative sources of waste other than the by-products of coal combustion [66].

It is widely known that foamed geopolymers can also be produced from available and chemically stable raw materials such as metakaolin. However, this can involve higher costs. If foamed geopolymer products based on metakaolin are produced on a mass scale, they will not be price competitive compared to organic materials and wool. The average cost of 1 ton of metakaolin in recent years has been about 400 USD. When considering specialist applications, this is not an exorbitant amount, but it can be a significant problem in the case of mass production which will probably limit the possibility of using geopolymers in certain areas where cost-effective solutions are sought.

Another issue is related to changes in the prices of raw materials, especially sodium hydroxide. Significant fluctuations in its price have been observed over the past several years. It should be remembered that sodium hydroxide (and aqueous solution of sodium silicate) is one of the main components of the alkaline solution used for the production of geopolymers.

During the global recession in 2008–2009, the price of caustic soda skyrocketed to over 800 USD/dmt [67]. Although this price effect was short-lived, it affected the industries that use caustic soda in production. The current prices of this material are quite lower, but significant price fluctuations are still noticeable.

Other problems related to the implementation of geopolymer technology on a mass scale are legislative and legal issues in many countries. This includes the need to obtain appropriate certificates, attestations, etc., which adds to the cost of implementation and is also considered as an obstacle that may discourage potential entrepreneurs interested in starting production.

Moreover, attention should be paid to the necessity of manufacturing an appropriate production line, the elements of which should be made of materials with suitable quality. Due to the nature of geopolymers, the materials used should be resistant to alkalis—above pH 14. This also leads to an increase in overall costs associated with the implementation of geopolymer technology.

Another major challenge is convincing the potential end-users of the new technology, which has not been used before. In many countries to date, materials produced using waste from the power industry or metallurgical industry have not enjoyed a good reputation as the technologies used in the 1970s and 1980s to produce them were of dubious quality. As a result, in Poland for example, it is difficult to convince the public that waste-based materials are harmless. This may also be a reason for lower interest in the production of such materials on a global scale.

The above-mentioned problems related to the implementation of new technology on mass scale can be possibly overcome; however, it will require a huge financial effort and is a great challenge for hundreds of scientists around the world.

Due to these reasons, it can be assumed that, for many years to come, waste-based materials including geopolymers will find applications only in niche areas such as in industrial installations as insulation materials.

### 3.2. Efflorescence—One of the Most Serious Problems

The main problems that must be overcome in the production of geopolymers are efflorescence and tarnishing on the surface of geopolymers, which destroy their structure as well as reduce their esthetic value. These cause the degradation of geopolymer plastics and concretes in a similar way as that of conventional concrete with Portland cement.

Efflorescence on the surfaces of geopolymers can be a critical challenge in their applications, although its effect on the mechanical and chemical stability of these materials has not yet been investigated. Several methods have been identified (including hydrothermal aging) that can effectively reduce efflorescence, but the mechanisms governing the mobility of alkali metal cations are not fully understood and require further studies [35]. Hydrothermal curing at elevated temperatures has been shown to reduce efflorescence on geopolymers. It was confirmed that a more suitable geopolymer binder with improved properties can be created by adding sufficient amounts of active alumina to precursors such as natural pozzolana, or by manipulating the curing conditions to increase the amount of alumina released from the less reactive phases of the precursor. Sodium aluminosilicate-based geopolymers, especially synthetic ones with high Na_2_O/Al_2_O_3_ ratios, can reveal unsightly efflorescence on the surface due to an excessive amount of sodium oxide, which is a residue of unreacted material. Sodium cations are mobile in the lattice pores, especially when there is moisture movement in the sample. One example is concrete in contact with soil moisture, where water moves upward through capillary forces, and then evaporates from the surface, leaving the alkaline cations present in the pores. Complex alkalis can react with CO_2_ in the atmosphere, resulting in the formation of white deposits (carbonates) on the surface, a condition known as efflorescence. Carbonation typically results in the degradation of binders, reduction of pH, and deposition of carbonate reaction products in the sample mass, which may or may not be visible to the naked eye, while efflorescence results in visible surface deposits which may or may not be accompanied by further degradation of binder. The efflorescence tendency in geopolymers is partly caused by the open microstructures of some materials, which are less reactive, and partly due to the high concentration of alkali metals in the pores, or relatively weak bonding (and exchangeability) of Na in the geopolymer structure. Some attempts have been made to reduce efflorescence with the use of potassium hydroxide instead of sodium hydroxide as an activator because potassium is more strongly bound in the aluminosilicate gel and potassium carbonate crystals tend to be less visible than sodium carbonate [68].

Some methods involve the annealing of geopolymers above 550 °C, i.e., above the temperature of NaOH decomposition. However, these are associated with increased costs and CO_2_ emissions. At present, despite the efforts of scientists and technologists around the world, this problem remains a challenge and reports indicate the withdrawal of products based on geopolymers from the market, mainly due to efflorescence (e.g., geopolymer grouts).

Thus, elimination of efflorescence is certainly a critical goal to be achieved for the introduction of geopolymer-based foam insulation materials.

## 4. Is There Anything We Can Do to Increase the Chance of Implementation?

The actions that need to be taken to increase the possibility of implementation of geopolymer technology on a mass scale are dependent on location and local conditions. It is certain that, all over the world, appropriate conditions should be created for companies interested in the implementation of geopolymer technology. There are many motivations for implementing this technology on a mass scale, and also for the use of geopolymers as foamed insulation materials.

**Figure 6:**
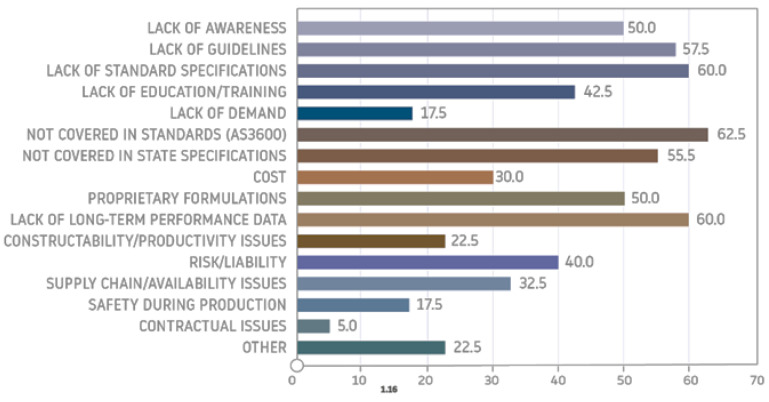
Respondents’ responses regarding barriers to geopolymer implementation [69].

A survey-based article [51] showed that the main motivation behind the search for alternative binders such as geopolymers is that the production of Portland cement consumes an enormous amount of energy and affects our environment. The respondents of the survey indicated that geopolymer cement is more environmentally friendly than Portland cement and has good performance properties. They also indicated that geopolymer may be qualified as a viable alternative to other binders, but it is expensive. The study however revealed that the majority of the respondents lacked knowledge about geopolymers and there is a need to engage experts, as well as organize training and seminars to increase public awareness about these materials. A review of the literature and industrial surveys [16] showed the issues that need to be addressed regarding the widespread implementation of geopolymers. The authors of the paper identified that it is most important to develop standard specifications, and new standards for geopolymer concrete to include performance requirements, ensuring the use of national and local specifications, and to perform more independent testing on engineering properties and long-term durability/resistance. They indicated that, in the short term, geopolymers will likely be used the most in precast and nonstructural applications and sidewalks, and as fire- and chemical-resistant materials (Figure 6).

One of the things we can do to increase the possibilities of geopolymer implementation is to increase our knowledge of producing stable and repeatable foams. One of the methods of better control over the production of stable foams can be a better understanding of their formation mechanisms and analytical descriptions using mathematical models.

The research results show that it is possible to successfully design a porous geopolymer with geometrically insulated rounded pores. The control of the percolation of the phases with the evolution of the pores allows their analytical description using models such as Maxwell–Eucken for a more homogeneous matrix [63].

## 5. Forecast and Summary

**Figure 7:**
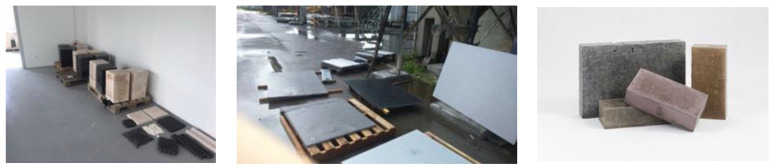
Examples of geopolymer products manufactured on a semi-technical scale in Poland (source—own research).

Some concerns have been raised about the difficulty of implementing geopolymer technology, especially in terms of insulation materials. Despite many attempts in countries around the world, this technology has not yet reached the level of implementation predicted several years ago. This state of affairs is due to several reasons described in this paper. In Poland, intensive work has been undertaken to implement geopolymer technology in many companies and scientific entities. Successful technical-scale tests have been carried out, but in the end the problems related to implementation prevented further development. Figure 7 presents selected geopolymer components manufactured on professional industrial lines and tested in real conditions.

There is no doubt that geopolymer technology will continue to develop intensively [70,71,72,73,74,75,76,77,78]. A key driver for the production of geopolymer concretes is the prospect of a significant reduction in CO_2_ emissions associated with their production process compared to the production of Portland cement. In recent years, large-scale demonstration projects, such as those carried out in Australia, have stimulated further basic research, especially studies on the durability of geopolymer concretes and engineering of production [13].

Geopolymers can be a viable alternative to concretes based on Portland cement mainly due to the reduced CO_2_ emissions (compared to OPC) [79,80]. The authors of [81] presented the results confirming that the CO_2_-e emission of geopolymer concrete is 9% less than OPC, despite the fact that many scientists previously claimed that it was a greater reduction value.

However, we should also pay attention to the prices, which may be even 30% [81] or three times higher for GP than for OPC [80]. The costs for foamed geopolymer materials will be similar to those for solid materials, but one should also remember about the use of foaming agent. The cost analysis show that the foaming agents are responsible for a small percentage of foam geopolymers total cost being that the alkaline activators are responsible for more than 80% [50]. (Issues related to the costs of raw materials have already been described in Section 3.1). There are various analyzes showing the advantages of geopolymeric materials in terms of environmental impact, but this is not a clear-cut issue. Many authors of works have divergent opinions on this subject. The most reasonable approach seems to be in line with the results of the LCA authors that geopolymeric binders have a similar impact on the natural environment as traditionally used binders [82,83].

**Figure 8:**
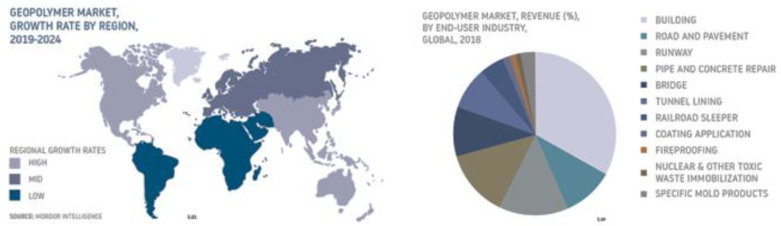
Geopolymer Products Market Forecast from 2019 to 2024 (source: Mordor Intelligence—online data).

The forecasts created by Mordor Intelligence (Figure 8) show that the use of geopolymers will increase worldwide. However, this applies to all the applications of geopolymers and not just as foamed materials. For solid materials, an increase in applications as an alternative to ready-mixed concrete in mass production is possible, whereas for foamed insulating materials, the outlook is not so optimistic due to a number of additional problems described in this article.

In recent years, the number of patents relating to geopolymers, both as binders and as foams, has been increasing intensively, which proves the increased interest in these materials and intensive work on the development of geopolymer technology. Patents application related to solutions allowing for the use of these materials as thermal insulation and fire protection are also worth mentioning [84,85,86].

One of the barriers to the implementation of geopolymers on a mass scale may also be the content of heavy metals and other compounds (TDS, sulphates and chlorides, fluorides) in the waste used for the production of geopolymers. The advantage is that geopolymers have the ability to immobilize many metals, but unfortunately in many cases, depending on the national standards allowing construction products, it can be a significant barrier.

Many authors in scientific studies show that compared to Portland cement technology, solutions for containment of hazardous waste in geopolymers and alkaline activated binders offer much greater possibilities of immobilizing heavy metals. Research on the possibility of immobilizing metals in alkali-activated binders has shown that alkali-activated slags can be used as an agent for the immobilization of heavy metals [64].

The results showed that the leaching of metal in a geopolymer matrix based on fly ash from plant combustion and volcanic ash was significantly reduced. Leaching levels were significantly below the limit set by the United States Environmental Protection Agency (USEPA) [65].

The issues related to the leaching of heavy metals should not be a major barrier to the implementation of geopolymers (this is an advantage for geopolymers compared to other technologies).

**In summary**, the most significant barriers to the widespread use of geopolymers include the following:

1. The basic materials for geopolymer production, such as fly ash originating from coal combustion and smelter slag, are not widely available in many countries, and are often treated, according to the law, as industrial waste and not as a raw material for the production of materials such as those used in construction (with some exceptions). Other raw materials such as metakaolin are too expensive compared to cement.
2. For about 50 years, there has been rapid development of admixtures, including polymeric ones, which are used to improve the setting processes in Portland concretes. However, these affect the strength of concretes and other properties such as water absorption, frost resistance, and abrasion resistance. Such additives are not yet commercially available for geopolymers. Additives used for Portland cement-based concretes do not work well for geopolymer concretes, and so further research is needed.
3. Portland cement-based concretes have been used since 160 years ago, and experience in their use can be considered comprehensive. On the other hand, the number of practical applications of products and structures is very limited in the case of geopolymer concretes. The current procedures used for the design of concrete structures and products are based on the relationships associated with the behavior of the Portland cement-based concrete structures under various conditions of loading and service environment. These relationships cannot be uncritically transferred to geopolymer concretes, and the existing range of research on the properties of these concretes also does not allow the development of new design procedures.
4. Most of the issues associated with Portland cement-based concretes are standardized. The developed standards for Portland cement-based concretes start from the assumption that Portland cement is the primary binding component, even when additives such as fly ash or blast furnace slag are used to produce these concretes. By contrast, the issues related to geopolymer concretes are standardized in only a few countries, which constitutes a key barrier for the widespread application of these materials.

Taking into account the arguments for and against the possibility of the mass production of geopolymer insulating materials, it should be stated that due to a number of problems related to the stability of foams and repeatability of parameters in mass production, as well as high sensitivity of the technology to changes in the prices of raw material, the mass production of foamed geopolymer insulating materials will not be commenced within the next few years. Significant steps should be made in research on this type of material by scientists and also to produce geopolymer materials that are activated not only by sodium hydroxide but also by acids. However, it is possible to use foamed geopolymers on a small scale for niche applications, which is already performed in several countries, but the challenge in achieving the performance of commonly used insulation materials, such as expanded polystyrene, polyurethane foams, or cellulose fibers, remains open.

The search for other alternative sources of raw materials for the production of geopolymers is another major task. In many parts of the world, due to environmental policies and the abandonment of the use of coal in power generation, the production of geopolymers from coal combustion by-products has become impractical.

## Data Availability

The data presented in this study are available on request from the corresponding author.
